# Unraveling Homologous Recombination Deficiency in Ovarian Cancer: A Review of Currently Available Testing Platforms

**DOI:** 10.3390/cancers17111771

**Published:** 2025-05-25

**Authors:** Nicola Marconato, Orazio De Tommasi, Dino Paladin, Diego Boscarino, Giulia Spagnol, Carlo Saccardi, Tiziano Maggino, Roberto Tozzi, Marco Noventa, Matteo Marchetti

**Affiliations:** 1 Department of Women and Children’s Health, University of Padua, 35128 Padua, Italy; nicola.marconato@phd.unipd.it; 2 Unit of Gynecology and Obstetrics, Department of Women and Children’s Health, University of Padua, 35128 Padua, Italy; 3 AB ANALITICA S.r.l., Via Svizzera, 16, 35127 Padova, Italy; paladin@abanalitica.it (D.P.); boscarino@abanalitica.it (D.B.)

**Keywords:** advanced epithelial ovarian cancer, homologous recombination deficiency, poly-ADP-ribose polymerase inhibitors, homologous recombination repair, test

## Abstract

Homologous recombination deficiency (HRD) is a key biomarker associated with increased sensitivity to PARP inhibitors (PARPi) in advanced epithelial ovarian cancer. Accurate identification of HRD status is essential for selecting patients most likely to benefit from these therapies. Current diagnostic approaches combine sequencing to detect mutations in homologous recombination repair genes—particularly BRCA1 and BRCA2—with genome-wide analysis of structural genomic alterations indicative of HRD. This review briefly outlines the biological basis of HRD and its clinical significance and then focuses on currently available assays for HRD assessment. We compare their molecular strategies, including the use of targeted gene panels and genomic instability metrics such as loss of heterozygosity, telomeric allelic imbalance, and large-scale state transitions. The review also highlights the strengths and limitations of each platform and discusses their role in guiding clinical decision-making. Challenges related to dynamic tumor evolution and the interpretation of HRD status in recurrent disease settings are also addressed.

## Simple Summary

Homologous recombination deficiency (HRD) is associated with increased sensitivity to PARP inhibitors (PARPi), which offer improved progression-free survival (PFS) in HRD-positive patients compared to those with homologous recombination proficiency (HRP). Access to PARPi therapy requires confirmation of HRD status, typically determined through tumor DNA sequencing to identify BRCA1/2 mutations or specific patterns of genomic instability indicative of HRD. Several diagnostic assays are available to assess HRD, employing different approaches—either through targeted gene panels or genome-wide analysis. This review focuses on the biological basis and consequences of HRD, the clinical use of PARPi, and the currently available diagnostic tools for evaluating HRD status.

## 1. Introduction

Ovarian cancer is the eighth most common malignancy and a leading cause of cancer-related death among women worldwide. In 2020, it accounted for 3.7% of all cancer cases and 4.7% of cancer deaths [1]. The cornerstone of ovarian cancer treatment is the combination of extensive multivisceral cytoreductive surgery—aimed at achieving no residual disease—and platinum-based chemotherapy. However, significant improvements in survival outcomes have been achieved through the development of novel targeted maintenance therapies [2,3].

**Figure 1:**
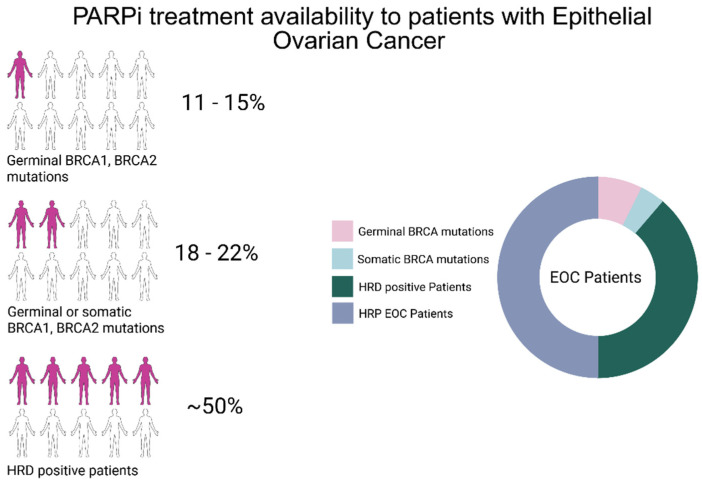
Schematic representation of patients with advanced epithelial ovarian cancer eligible for PARPi treatment. Around 11–15% of EOC patients harbor germline deleterious mutations in BRCA1 or BRCA2. Around 18–22% of EOC patients harbor somatic or germinal BRCA1 or BRCA2 mutations. The introduction of HRD tests that analyze the genome using genome-wide strategies has helped to increase patients eligible for PARPi treatment to around 50% [7]. This image has been created with BioRender.com.

Poly (ADP-ribose) polymerase inhibitors (PARP inhibitors, PARPi) were the first targeted therapies approved for ovarian cancer [4]. In the phase III SOLO1 study, maintenance treatment with olaparib in patients with deleterious germline BRCA1/2 mutations significantly prolonged progression-free survival (PFS): 56.0 months compared to 13.8 months with placebo [5,6]. To extend these benefits to patients with somatic or non-germline BRCA mutations, the ENGOT-OV16/NOVA trial tested niraparib against placebo. Median PFS in BRCA-mutated patients was 21.0 months with niraparib compared to 5.5 with placebo. The PAOLA-1/ENGOT-ov25 study further demonstrated that patients with high genomic instability due to homologous recombination deficiency (HRD) also derived significant benefit from PARPi, effectively expanding treatment eligibility from 18–22% to nearly 50% of patients (Figure 1) [7]. These findings led to the development of new HRD assays aimed at improving sensitivity, specificity, and cost-effectiveness. HRD testing helps identify patients who are most likely to benefit from PARPi therapy, while sparing homologous recombination proficient (HRP) patients from unnecessary toxicity and healthcare costs [8].

This review focuses on currently available diagnostic assays for assessing HRD in ovarian cancer. We provide a comparative overview of their molecular basis, analytical features, clinical validation, and regulatory status. A brief background on homologous recombination and the rationale for PARPi sensitivity is also included to contextualize the clinical utility of HRD testing.

## 2. Background and Rationale

### 2.1. Homologous Recombination Repair Mechanism

**Figure 2:**
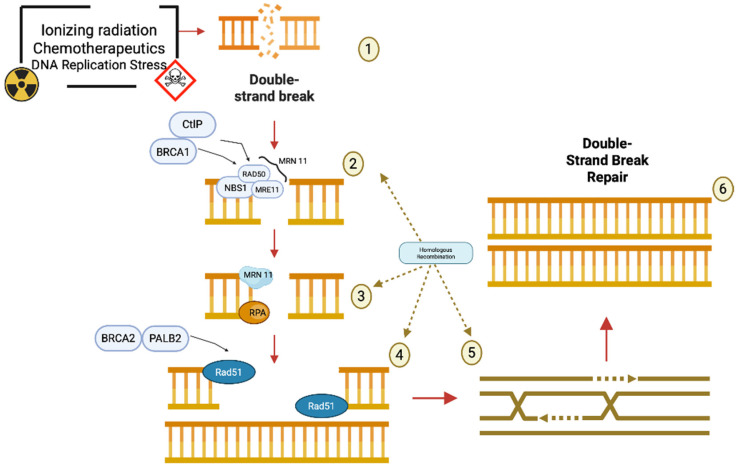
Schematic representation of the homologous recombination repair pathway to repair double-strand breaks, red arrows describe the flow of events during Homologous Recombination, brown dashed arrows underline that steps 2-5 belongs to the Homologous Recombination mechanism: (**1**) Double-strand breaks (DSBs) in DNA can be caused by various damaging agents such as ionizing radiation, chemotherapeutic drugs, and replication stress. (**2**) Upon the occurrence of a DSB, the MRN complex—composed of MRE11, RAD50, and NBS1—is recruited to the site of damage. CtIP and BRCA1 assist this complex in the resection of the DNA ends to produce 3′ single-stranded overhangs. (**3**) The resulting single-stranded DNA is immediately coated by the RPA protein, which protects it from degradation and prevents the formation of secondary structures. The MRN complex continues to participate in processing. (**4**) BRCA2 and PALB2 then facilitate the replacement of RPA with RAD51, enabling the formation of a RAD51 nucleoprotein filament on the single-stranded DNA. (**5**) RAD51 promotes the search for a homologous DNA sequence and mediates strand invasion, initiating homologous recombination. This step ensures that the correct genetic information is used as a template for repair. (**6**) The outcome of this coordinated repair process is the accurate double-strand break repair through homologous recombination, leading to the restoration of the original DNA structure and the preservation of genomic integrity. This image has been created with BioRender.com.

Cells are constantly exposed to sources of DNA damage, including double-strand breaks (DSBs), which are among the most cytotoxic lesions. To maintain genomic stability, cells rely on several repair mechanisms. Non-homologous end joining (NHEJ) and microhomology-mediated end joining (MMEJ) are fast but error-prone pathways that often lead to insertions, deletions, or chromosomal rearrangements. In contrast, homologous recombination repair (HRR) is a high-fidelity pathway that uses the sister chromatid as a template, ensuring accurate repair and preserving genome integrity [9]. HRR is primarily active during the S and G2 phases of the cell cycle, when a sister chromatid is available. The process begins with 5′ to 3′ resection of DSB ends by the MRN complex (MRE11–RAD50–NBS1), assisted by BRCA1 and CtIP, generating 3′ single-stranded DNA (ssDNA) overhangs (Figure 2) [10]. These overhangs are initially coated by replication protein A (RPA) to protect them from degradation and secondary structure formation [11].

BRCA2, with the help of PALB2 and DSS1, facilitates the replacement of RPA by RAD51, which polymerizes along the ssDNA to form a nucleoprotein filament [12]. This filament is essential for locating the homologous sequence on the sister chromatid and initiating strand invasion. The RAD51 nucleoprotein filament performs the homology search and strand invasion, forming a displacement loop (D-loop) [13]. DNA synthesis is then carried out using the homologous strand as a template. Subsequently, DNA polymerases synthesize new DNA, restoring the original sequence. Depending on the context, different subpathways complete the repair process, resulting in accurate restoration of the DNA sequence [14].

The process is tightly regulated and highly coordinated, involving several co-factors such as RAD51 paralogs and helicases that stabilize the recombination intermediate and facilitate proper resolution. Disruption of any key component in this pathway can lead to defective HRR, resulting in genomic instability—a hallmark of many cancers [15]. Given its central role in preserving genomic integrity, HRR has become a critical target in oncology. Loss-of-function mutations in HRR-related genes, especially BRCA1 and BRCA2, sensitize tumor cells to DNA-damaging agents and PARPi, providing the biological rationale behind HRD testing in clinical practice.

### 2.2. Homologous Recombination and PARP Inhibition

As previously described, HRR is a high-fidelity mechanism that ensures the accurate repair of DSBs through the use of a sister chromatid. The key steps of this process—including DNA end resection, RAD51-mediated strand invasion, and template-driven synthesis—are essential for preserving genomic integrity. When this pathway is disrupted, cells become reliant on alternative, error-prone repair mechanisms, creating a therapeutic vulnerability that can be exploited through PARP inhibition [16].

PARP plays a central role in the cellular response to DNA damage by facilitating repair and maintaining genomic stability. PARP is involved in single-strand breaks DNA repair, and PARP loss of function significantly impairs the ability of the cell to repair single-strand breaks. PARP inhibition leads to accumulation of unrepaired single-strand DNA lesions due to PARP being stalled on the damage site. The accumulation of ssDNA breaks after several cell cycles leads to cell cycle arrest at the G2 M checkpoint, ultimately resulting in apoptosis, especially in cells under replicative stress [17,18].

In cells with HRR defects, such as those with BRCA1 or BRCA2 mutations, this deficiency triggers a state of synthetic lethality. Unrepaired SSBs stall replication forks and collapse into DSBs, which HRR-deficient cells cannot resolve, resulting in cell death. Synthetic lethality through PARP inhibition can also be explained by the gap model: Another function of PARP1 is to mend unligated Okazaki fragments during lagging-strand maturation. When PARP is inhibited, it becomes stalled at these sites, depleting the pool of available replication protein A (RPA) and RAD51, leading to the formation of unprotected ssDNA gaps that can evolve into double-strand DNA breaks, which cannot be repaired if BRCA genes are not functional. PARPi sensitivity can also be acquired in this model through mutation in proteins belonging to the Okazaki fragments, such as flap endonuclease I (FEN1), LIG1, XRCC1, or proliferating cell nuclear antigen (PCNA) [17,18].

Preclinical studies have shown that BRCA1- and BRCA2-deficient cells exhibit a 57-fold and 133-fold increase in sensitivity to PARPi, respectively. Additionally, loss of 53BP1 can partially restore HRR activity in BRCA1-deficient cells, potentially influencing PARPi sensitivity and resistance. This supports its investigation as a predictive biomarker in BRCA-mutant and triple-negative breast cancers [19].

PARPi are NAD^+^ analogs that competitively inhibit PARP’s catalytic activity, blocking poly (ADP-ribose) chain formation and preventing recruitment of repair proteins. Beyond catalytic inhibition, their cytotoxicity is also mediated by their ability to trap PARP-DNA complexes, interfering with replication fork progression [20]. The potency of PARP trapping varies among agents, with talazoparib showing the highest trapping efficiency, followed by niraparib, olaparib, rucaparib, and veliparib [21].

Together, these mechanisms explain the clinical rationale for using PARPi in HRR-deficient tumors and highlight the importance of accurately identifying HRD status in patients to guide treatment decisions.

### 2.3. Clinical Applications and Regulatory Approvals of PARP Inhibitors

The clinical benefit of PARPi is well established in ovarian cancer, where they are approved both for the treatment of recurrent disease and as maintenance therapy in patients who respond to platinum-based chemotherapy. The phase III SOLO2 [22] trial demonstrated that olaparib significantly prolonged PFS in patients with relapsed high-grade serous ovarian cancer carrying BRCA1/2 mutations. These findings were supported by additional trials, including SOLO3 [23] and OlympiAD [24], confirming olaparib’s efficacy in germline BRCA-mutated ovarian and breast cancers, respectively.

Talazoparib showed similar benefits in the ABRAZO [25] and EMBRACA studies [26], improving PFS and quality of life compared to chemotherapy. Beyond gynecologic cancers, the POLO [27] and PROfound trials [28] expanded the indication of PARPi to pancreatic and prostate cancers with BRCA mutations or broader HRD phenotypes.

Olaparib received accelerated FDA approval in 2014 for BRCA-mutated cancers, and its role was later established in recurrent ovarian cancer based on SOLO2 trial [22], leading to its approval as maintenance therapy following platinum response. The SOLO3 study [23], however, failed to demonstrate an overall survival (OS) benefit in the treatment setting, resulting in the withdrawal of its indication for relapsed disease by the FDA in 2022. In the first-line setting, the SOLO1 trial [5] showed significant benefit for BRCA-mutated patients, while the PAOLA-1 study [29] expanded its indication to include HRD-positive patients when combined with bevacizumab. Niraparib followed a different regulatory path. The NOVA trial [30] supported its approval in 2017 as maintenance therapy for recurrent platinum-sensitive disease, independent of BRCA status. It was later approved for first-line maintenance in 2020, based on the PRIMA study, which demonstrated benefit in both BRCA-mutated and HRD-positive patients, and to a lesser extent in the overall population. Additionally, the single-arm QUADRA trial [31] supported its use in heavily pretreated HRD-positive patients, although this did not lead to a broad treatment indication. Rucaparib received initial FDA approval in 2016 based on early-phase trials (ARIEL2 [31], Study 10 [32]) in the recurrent setting, and was granted full approval following the ARIEL3 trial [33], which confirmed its efficacy as maintenance after platinum response. However, results from study [34] showed limited OS benefit, prompting withdrawal of its treatment indication in 2022. Finally, the ATHENA-MONO trial recently contributed evidence supporting the use of rucaparib in the first-line maintenance setting, particularly in patients with HRD-positive tumors, though regulatory decisions are still evolving.

These regulatory shifts underscore the importance of distinguishing between first-line and recurrent settings when evaluating PARPi trials. They also highlight the growing role of HRD testing in selecting patients most likely to benefit from PARP inhibition across different stages of treatment.

### 2.4. Homologous Recombination Deficiency

Homologous recombination deficiency refers to the impaired function of genes involved in the HRR pathway. While several genes contribute to HRR—including BRCA1, BRCA2, RAD51, PALB2, ATM, ATR, and others—BRCA1 and BRCA2 remain the most prominent, both in terms of clinical testing and therapeutic response to PARPi [35].

BRCA1/2 mutations can be germline or somatic and typically result in truncated, nonfunctional proteins. Epigenetic silencing, particularly BRCA1 promoter hypermethylation, can also lead to functional loss. Together, these alterations define a major subset of HRD-positive tumors. BRCA2 exon 11, for example, encodes the BRC domain that is critical for RAD51 interaction, and it is a frequent mutational hotspot. Beyond BRCA genes, mutations in other HRR genes have been associated with an HRD phenotype and may predict sensitivity to platinum-based chemotherapy and PARPi. However, their clinical impact remains less well defined, and their correlation with HRD is often incomplete [36].

At the genomic level, HRD manifests through a set of structural alterations collectively known as “genomic scars”, which result from error-prone DNA repair processes such as NHEJ and MMEJ. The most widely recognized genomic scar features include the following:

- Loss of heterozygosity (LOH): the loss of one parental allele across chromosomal regions.
- Telomeric allelic imbalance (TAI): unequal allele representation near telomeres.
- Large-scale state transitions (LST): chromosomal breaks generating DNA segments ≥10 Mb.

These features can be quantified using SNP arrays or next-generation sequencing, and they form the basis of many commercial HRD assays [37]. Copy number variations (CNVs), single nucleotide variants (SNVs), and small insertions/deletions (indels) further contribute to HRD-associated mutational profiles. In addition to structural metrics, specific mutational signatures have been associated with HRD. Among these, mutational signature 3—characterized by the pattern of single base substitutions and indels associated with BRCA loss—is the most validated. Whole genome or exome sequencing (WGS/WES) can also identify rearrangement signatures and composite patterns that improve HRD classification [38].

Despite the presence of HRD markers, not all tumors respond to PARPi. Resistance may emerge through reversion mutations that restore HRR gene function, or through epigenetic changes, such as BRCA1 promoter demethylation, that reactivate gene expression. Additional mechanisms include activation of alternative DNA repair pathways or increased drug efflux, which reduce intracellular drug levels. Notably, resistance does not eliminate the HRD genotype; tumors retain genomic scars and thus the HRD positivity upon retesting, while acquiring resistance to PARPi, particularly in the recurrent setting. This limitation complicates the interpretation of HRD assays in previously treated patients [39].

Another clinically relevant biomarker is CCNE1 amplification, which is commonly associated with HRP. High CCNE1 copy number correlates with enhanced DNA repair capacity and poor response to PARPi [40,41].

In summary, HRD is a central biomarker in ovarian and other cancers, encompassing both genetic alterations and genomic instability patterns. Accurate characterization is essential for predicting therapeutic response and guiding the use of PARPi and other DNA-damaging agents [42].

### 2.5. Diagnostic Strategies for HRD Assessment

Current ESMO guidelines recommend testing for somatic BRCA1 and BRCA2 mutations as part of the HRD assessment. Deleterious alterations in these genes are consistently associated with improved PFS in patients treated with PARPi [43]. In contrast, pathogenic variants in other HRR-related genes show a less robust correlation with PARPi sensitivity [44]. To expand HRD detection beyond BRCA1/2 mutations, several genome-wide approaches have been developed. These strategies focus on identifying patterns of genomic instability that characterize HRD-positive tumors, aiming to predict benefit from PARPi therapy even in the absence of detectable BRCA mutations. The underlying concept is that the extent of genomic instability may serve as a surrogate for HRR deficiency.

The first FDA-approved assay in this category was myChoice^®^ CDx (Myriad Genetics), validated in the PAOLA-1 trial. This platform combines three metrics—LOH, TAI, and LST—into a single HRD score derived from genome-wide SNP analysis. Tumors with scores above a defined threshold are classified as HRD-positive [45,46]. Another commonly used platform is FoundationOne^®^ CDx (Foundation Medicine), which includes somatic BRCA mutation testing and computes genomic LOH (gLOH) based on genome-wide copy number profiles and allele frequencies. It covers 324 genes and was approved for BRCA mutation detection in trials like SOLO-1 [6,47]. A more recent and cost-effective strategy is low-pass whole genome sequencing (shallow WGS), used in platforms such as SeqOne HRD. This method evaluates somatic CNVs across the genome and calculates an HRD score based on large-scale instability patterns [48].

While these tests represent important advances, they all share a key limitation: they assess the downstream consequences of HRD—so-called genomic scars—rather than real-time HRR functionality. Tumors that were historically HRD-positive may reacquire DNA repair capacity through reversion mutations or epigenetic reactivation, potentially resulting in PARPi resistance despite a positive test. Consequently, some HRP tumors may be misclassified as HRD-positive and do not benefit from treatment [49].

## 3. Available Assays for HRD Analysis

Following the clinical and regulatory validation of assays like Myriad’s myChoice CDx and FoundationOne CDx, several platforms have emerged to enhance the detection of homologous recombination deficiency. These assays, developed by commercial entities, healthcare institutions, or academic groups, aim to refine HRD evaluation through improved analytical methods and broader genomic coverage [45,46,47].

The following sections outline the principal assays currently in clinical or investigational use, highlighting their methodologies, gene panels, and genome-wide strategies for detecting HRD-associated genomic instability (Table 1).

### 3.1. MyChoice CDx, Myriad Genetics

MyChoice CDx was the first HRD test approved by both the FDA and EMA, following its validation in the PAOLA-1 clinical trial. The assay is performed centrally at Myriad’s laboratory using formalin-fixed, paraffin-embedded (FFPE) tumor tissue. The test integrates BRCA1 and BRCA2 gene sequencing with a genome-wide SNP microarray that analyzes approximately 54,000 single nucleotide polymorphisms (SNPs). From these data, it derives three indicators of genomic instability: loss of heterozygosity (LOH), telomeric allelic imbalance (TAI), and large-scale state transitions (LST), which are then combined into a genomic instability score (GIS). A tumor is classified as HRD-positive if it either harbors a deleterious mutation in BRCA1 or BRCA2 or if the GIS exceeds a predefined threshold of 42. Based on the findings from the PAOLA-1 trial, myChoice CDx serves as the companion diagnostic for the use of olaparib in combination with bevacizumab in patients with ovarian cancer [50].

### 3.2. FoundationOne CDx, Foundation Medicine

FoundationOne CDx received FDA approval as a companion diagnostic to identify ovarian cancer patients eligible for olaparib monotherapy, based on findings from the SOLO-1 trial. The assay is performed at Foundation Medicine’s central laboratory on formalin-fixed, paraffin-embedded (FFPE) tumor tissue. The assay sequences a comprehensive 324-gene panel associated with multiple cancer types. It detects a wide range of genomic alterations, including single nucleotide variants (SNVs), insertions and deletions (indels), copy number variations (CNVs), genomic rearrangements, tumor mutational burden, microsatellite instability, and the percentage of genomic loss of heterozygosity (gLOH). Tumors harboring somatic mutations in BRCA1 or BRCA2 are considered potentially sensitive to PARP inhibitor therapy with olaparib. In addition, gLOH is calculated from genome-wide copy number and allele frequency data. A gLOH score greater than 16 has been associated with improved progression-free survival (PFS) in patients receiving rucaparib maintenance therapy [47].

### 3.3. SOPHiA DDM Dx HRD Solution, SOPHiA GENETICS

The SOPHiA DDM Dx HRD Solution is a CE-IVD marked assay for the detection of homologous recombination deficiency (HRD), performed at SOPHiA GENETICS′ central laboratory using FFPE tumor samples. The analysis combines the sequencing of a 28-gene panel involved in the homologous recombination repair (HRR) pathway—including BRCA1 and BRCA2—with low-pass whole genome sequencing at an average depth of approximately 1x. These genome-wide data are processed by GIInger, a proprietary deep learning algorithm that calculates a genomic instability index (GII). The GII is used to estimate the tumor’s level of genomic instability and to predict the potential benefit from treatment with PARP inhibitors [51].

### 3.4. HRD Focus Panel, AmoyDx

The HRD Focus Panel by AmoyDx is a CE-IVD marked assay designed for the assessment of HRD status and is notable for its ability to be performed directly in research or clinical laboratories, without the need to send samples to a centralized facility. The assay analyzes FFPE tumor tissue, sequencing the entire coding regions and exon–intron boundaries of BRCA1 and BRCA2 genes.

In addition to BRCA sequencing, the test conducts a genome-wide analysis using approximately 24,000 single nucleotide polymorphisms (SNPs) distributed across the genome. These SNPs are processed using the GSS (Genomic Scar Score) algorithm, which evaluates multiple features of copy number alterations, including the length, type, genomic position, and number of breakpoints.

The final output quantifies the degree of genomic instability. If this exceeds a defined threshold, the tumor is classified as HRD-positive, suggesting that the patient may benefit from PARP inhibitor therapy in terms of improved progression-free survival (PFS) [52].

### 3.5. Oncomine Comprehensive Assay Plus, Thermo Fisher Scientific

The Oncomine Comprehensive Assay Plus by Thermo Fisher Scientific is a Research Use Only (RUO) test, distributed as a diagnostic kit for in-house laboratory use. The assay sequences a panel of 517 genes, including 46 genes involved in the homologous recombination repair (HRR) pathway, using DNA extracted from FFPE tumor tissue. Among the genes analyzed are BRCA1 and BRCA2. In addition to detecting somatic variants, the assay generates a genomic instability metric (GIM), which quantifies HRD-associated genomic scarring. The GIM score ranges from 0 to 100, with higher values indicating greater genomic instability. A GIM score ≥ 16 is considered indicative of a high genomic instability profile [53].

### 3.6. xT CDx, Tempus HRD, Tempus

xT CDx is a sequencing service developed by Tempus, performed in their central laboratory on FFPE tumor tissue. The assay targets 648 cancer-related genes, including intronic overhangs and selected promoter regions, along with 239 microsatellite instability (MSI) loci. Clinicians have the option to include the Tempus HRD module, which assesses HRD status using data derived from the same sequencing run. The analysis identifies deleterious BRCA1/2 mutations, evaluates BRCA1/2-specific loss of heterozygosity (LOH), and quantifies genome-wide somatic LOH. These data are integrated into a composite HRD score, which reflects the likelihood of a tumor responding to PARP inhibitor therapy [54,55].

### 3.7. TruSight Oncology 500 HRD, Illumina

The TruSight Oncology 500 HRD by Illumina is a Research Use Only (RUO) diagnostic kit that analyzes FFPE tumor DNA. The assay sequences approximately 500 cancer-related genes, including BRCA1 and BRCA2, and utilizes around 25,000 genome-wide probes to evaluate genomic instability. HRD status is calculated using the same algorithm implemented in Myriad Genetics’ myChoice CDx, combining three metrics—loss of heterozygosity (LOH), telomeric allelic imbalance (TAI), and large-scale state transitions (LST)—into a genomic instability score (GIS) [56,57].

### 3.8. SeqOne HRD, SeqOne Genomics

SeqOne HRD is a CE-IVDR marked assay for the evaluation of homologous recombination deficiency, available as both a diagnostic kit and a centralized testing service. The test analyzes FFPE tumor tissue from ovarian cancer patients. The workflow includes BRCA1 and BRCA2 targeted sequencing, combined with low-pass whole genome sequencing (lpWGS) to assess genome-wide copy number variations (CNVs). These CNVs are processed to compute two main genomic instability indicators: Large Genomic Alterations (LGA) and Loss of Parental Copy (LPC). Additionally, the test quantifies CCNE1 copy number, which has been associated with HR proficiency. All these parameters are integrated into a composite HRD score, used to predict sensitivity to PARP inhibitor therapy [48].

### 3.9. NOGGO GIS, Charite Berlin and University of Hamburg

NOGGO GIS is a Laboratory-Developed Test (LDT) for HRD detection, developed and performed at Charité Berlin in collaboration with the University of Hamburg. The assay is conducted on FFPE tumor samples and includes sequencing of BRCA1, BRCA2, and 55 additional genes involved in the homologous recombination repair (HRR) pathway. In addition to gene sequencing, the test analyzes approximately 20,000 genome-wide SNPs. These data are used to compute the NOGGO genomic instability score, which integrates three parameters: loss of heterozygosity percentage, copy number alterations percentage, and telomeric copy number alterations percentage. The optimal threshold for HRD positivity was established through comparative analysis with the Myriad myChoice CDx, with the highest concordance achieved at a cutoff between 83 and 85 [58].

### 3.10. Leuven HRD, University of Leuven

The Leuven HRD test is a Laboratory-Developed Test (LDT) performed at the University of Leuven, using FFPE tumor tissue. The assay sequences a panel of genes involved in homologous recombination repair, including BRCA1, BRCA2, RAD51C, RAD51D, PALB2, BLM, BARD1, BRIP1, and TP53. In addition to gene sequencing, the test includes genome-wide analysis of approximately 90,000 single nucleotide polymorphisms (SNPs). These SNPs are used to identify loss of heterozygosity (LOH), telomeric allelic imbalance (TAI), and large-scale transitions (LST), which are then combined to generate a genomic instability score (GIS). The tumor is considered HRD positive if the GIS is above 56. In a comparative analysis using ovarian cancer samples from the PAOLA-1/ENGOT-ov25 trial, Leuven HRD demonstrated a 91% overall concordance with the Myriad myChoice CDx test [59].

### 3.11. Hospital Geneva HRD Test, Geneva University

The Geneva HRD Test is a Laboratory-Developed Test (LDT) performed at the University Hospital of Geneva. The analysis is conducted on FFPE tumor tissue from ovarian cancer samples. The test focuses exclusively on BRCA1 and BRCA2 gene sequencing, alongside the evaluation of genome-wide copy number alterations (CNAs). From these data, it calculates a normalized large-scale transition (LST) score, with a defined threshold of 15 used to identify HRD-positive tumors. In a comparative study against Myriad myChoice CDx, the Geneva HRD Test demonstrated a positive agreement of 98% and a negative agreement of 81% [60].

## 4. Comments

Since the introduction of HRD testing, the proportion of patients eligible for PARP inhibitor (PARPi) therapy has increased from approximately 18–22% to nearly 50% [7]. Although this represents a major step forward in personalizing treatment, several critical issues persist regarding the implementation, interpretation, and equity of these diagnostic tools.

A central concern is accessibility. Most validated HRD assays—such as myChoice CDx, FoundationOne CDx, and SOPHiA DDM—require FFPE tumor samples to be shipped to centralized laboratories, which adds logistical complexity, processing time, and costs. The use of FFPE also introduces the risk of DNA degradation and artifacts, which may negatively impact sequencing accuracy and variant interpretation.

This issue is exacerbated in low- and middle-income countries, where HRD testing is often unavailable or prohibitively expensive. The use of Lab-Developed Tests (LDTs), such as those implemented by academic centers like Charité Berlin (NOGGO GIS) or the University of Leuven (Leuven HRD), might offer more flexible and scalable alternatives. However, LDTs require robust validation infrastructure and local expertise, which are not uniformly available across all healthcare systems. The broader adoption of CE-IVD marked kits, such as the AmoyDx HRD Focus Panel, could improve decentralization, but even these require sophisticated genomic facilities.

In several African regions, performing the genome-wide HRD test is unaffordable. One proposed alternative is genotyping three common deleterious mutations in BRCA genes, but the high cost of PARP inhibitors for ovarian cancer treatment remains a major barrier [61]. Regulatory disparities further limit access. Currently, myChoice CDx is the only FDA-approved genome-wide HRD assay, whereas FoundationOne CDx is limited to detecting BRCA1/2 mutations and recommending rucaparib based on gLOH status. Patients in the U.S. are thus excluded from using promising newer platforms, such as SeqOne, xT Tempus, or the Oncomine Comprehensive Assay, which may offer broader genomic profiling or improved sensitivity and specificity at lower costs.

**Figure 3:**
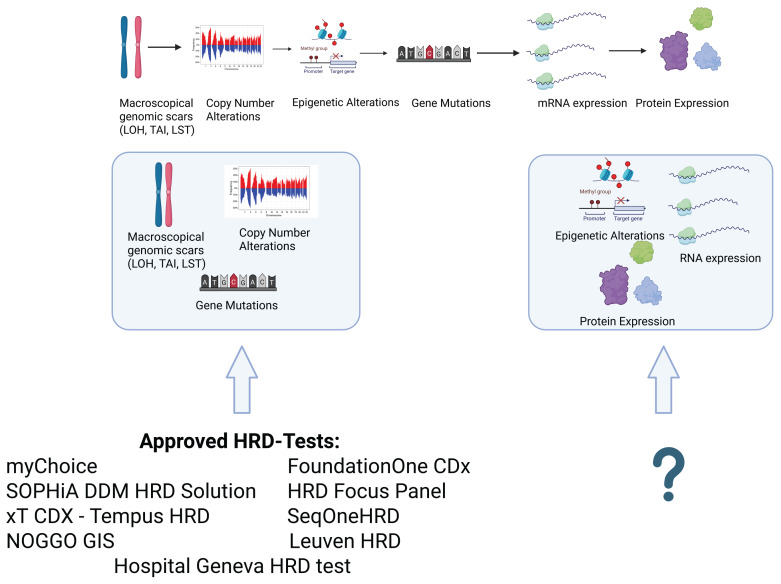
Commercially available tests mainly cover macroscopic genomic scars, copy number alterations, and mutations in their gene panels. No currently available or approved HRD test covers epigenetic alterations, mRNA expression, or protein expression. The inclusion of these features could improve the prediction of the emergence of PARPi resistance and broaden the availability of PARPi therapy to patients deemed HRD-negative. This image has been created with BioRender.com.

Another key issue is harmonization across tests. The cutoffs for HRD positivity vary widely (Table)—from a GIM ≥16 in Oncomine, to a GIS ≥83 in NOGGO GIS, to an HRD score ≥ 42 in myChoice CDx—making cross-platform comparisons difficult and potentially affecting treatment decisions. Furthermore, most of these tests still rely heavily on static genomic “scars”, which do not reflect dynamic changes in tumor biology, particularly in the context of acquired resistance. Emerging data support the integration of broader HRR gene panels (e.g., RAD51C/D, PALB2, ATM) and functional assays to increase the predictive value of testing and reduce false-positive rates. These additions could better distinguish truly HR-deficient tumors from those that harbor mutations without functional consequences (Figure 3). Importantly, they may also help identify patients with HRD-positive tumors who are unlikely to respond to PARPi due to secondary reversion mutations or replication fork stabilization mechanisms [62].

Despite these limitations, the rapid evolution of HRD diagnostics continues to improve treatment selection. The next frontier will require expanding decentralized, cost-effective, and clinically validated platforms, standardizing cutoffs and scoring systems, and incorporating functional and resistance-related biomarkers to ensure broader access and precision in therapeutic decision-making.

## 5. Future Perspectives

Future developments in HRD testing should aim not only to improve sensitivity and specificity but also to reduce costs and broaden global accessibility. One promising avenue is the use of shallow whole genome sequencing (sWGS), a cost-effective alternative to deep WGS that retains the ability to detect genome-wide features of genomic instability. An example is the ShallowHRD v2 software, which demonstrated a 94% concordance with Myriad myChoice CDx in the phase III PAOLA-1/ENGOT-ov25 trial [63,64].

At present, no HRD test can prospectively determine whether a tumor will exhibit primary or acquired resistance to PARP inhibitors. Future assays should address this limitation by incorporating biomarkers of resistance, such as BRCA reversion mutations or transcriptional signatures associated with functional restoration of homologous recombination. New tests should also analyze genes involved in the newly discovered gap model and in Okazaki fragment processing, such as FEN1, LIG1, XRCC1, and PCNA, which could also help to explain the emergence of PARPi resistance in those cases that could not be explained before the discovery of the gap model [17,18,62].

Another major area of interest is the development and clinical validation of functional assays, particularly those assessing RAD51 nuclear foci formation. RAD51 plays a key role in HRR by facilitating strand invasion at double-strand breaks (DSBs), working downstream of BRCA1 and BRCA2 [13]. Upon DNA damage, RAD51 accumulates at DSBs in coordination with BRCA2, forming subnuclear foci visible via immunofluorescence. The absence or reduction of these foci reflects a functionally deficient HRR system, independent of the underlying molecular cause [65].

Despite their potential, RAD51-based functional assays have limitations. They fail to identify tumors that are sensitive to PARP inhibitors due to defects in DNA repair pathways outside the canonical HRR machinery—such as alterations in ATM, RNaseH2, XRCC1, or ALC1—and are also unable to detect resistance mechanisms not involving HRR restoration, including replication fork stabilization [66,67].

Nevertheless, RAD51 foci analysis could play a valuable role in identifying patients with germline BRCA1/2 or PALB2 mutations whose tumors, despite their genetic background, are functionally HRR-proficient and therefore resistant to PARPi. Conversely, it may also help to identify patients without known mutations who still display a functional HRD phenotype, potentially making them good candidates for PARPi therapy [66].

In summary, the future of HRD diagnostics lies in integrating genomic, functional, and resistance-informed approaches to better stratify patients and guide therapy. Technological advancements such as sWGS, AI-driven algorithms, and RAD51-based assays represent promising steps toward more accessible, predictive, and clinically meaningful HRD testing.

## 6. Conclusions

The FDA approval of Myriad Genetics’ myChoice CDx following the PAOLA-1 trial marked a pivotal moment in the evolution of HRD diagnostics. This milestone has stimulated the development of numerous alternative and complementary assays aimed at improving detection accuracy and reducing associated costs. Leading diagnostics companies, as well as academic institutions, have launched new solutions to refine patient stratification and personalize therapy, particularly in the context of PARP inhibitor (PARPi) treatment for ovarian and breast cancer.

Although a wide range of HRD tests are now available, a significant need remains to improve their accuracy, reproducibility, and economic feasibility. The future of HRD diagnostics should prioritize reducing false positives, predicting therapeutic resistance, and minimizing inter- and intra-sample variability in HRD scores. Only through such efforts can HRD testing fully support the goals of precision oncology and equitable patient care.

## Figures and Tables

**Table 1:** Available HRD tests. The table reports the commercial name, owner name, gene panel sequenced, genome-wide method used, test type, and type of approval.

Commercial Name	Developer	Gene Panel	Genome-Wide Method Used to Calculate Genomic Instability	Algorithm	HRD Threshold	Average Turn-Around Time	FFPE Tissue Requirements	Reimbursement Status	Type of Approval
myChoice CDx	MYRIAD GENETICS	BRCA1, BRCA2	Analysis of 54,000 SNPs interspersed in the genome; they are used to calculate the genomic instability score	GIS (LOH + TAI + LST)	≥42	14 days	5 × 5 mm or 5–11 unstained slides, with >20% tumor cellularity	FDA reimbursed	CE-IVD, FDA approved
FoundationOne CDx	Foundation Medicine	324 genes, BRCA1 and BRCA2 included	Genomic loss of heterozigosity is calculated from the sequencing data	gLOH	≥16	8.8 days	1 mm^3^ either a block or 10 unstained slides, for both H&E slide is required	Reimbursed by Original Medicare and Medicare Advantage	CE-IVD, FDA approved
SOPHiA DDM HRD Solution	SOPHiA Genetics	28 genes, including BRCA1 and BRCA2	Low-pass whole genome sequencing data is analyzed by a deep learning based algorithm to determine a genomic instability index.	GII (AI algorithm: GIInger)	Proprietary threshold	Not available	Not available	Not available	CE-IVD
HRD Focus Panel	AMOY DX	BRCA1, BRCA2	24,000 SNPs are analyzed to determine the genomic instability.	GSS	Threshold by algorithm	3 days	Not available	Not available	CE-IVD
Oncomine Comprehensive Assay Plus	THERMO FISHER SCIENTIFIC	517 genes; 46 of them belong to the HRR pathway	The analysis of the gene panel returns the genomic instability metric.	GIM	≥16	3 days	>20 ng of DNA	No	Not approved
xT CDx + Tempus HRD	TEMPUS	648 genes	The analysis of the gene panel allows the determination of an HRD score.	Composite HRD score	Not disclosed	Not available	>50 ng of DNA	Reimbursed	LDT
TruSight Oncology 500 HRD	Illumina	523 genes and 55 RNAs	Genomic instability score is calculated through the same Myriad myChoice algorithm, which uses data from 25,000 probes.	GIS (same as Myriad)	≥42	Not available	2 mm^3^	No	Not Approved
SeqOne HRD	SeqOne Genomics	BRCA1, BRCA2 and CCNE1 copy number profile	It analyzes CNVs through sWGS; from them it calculates the LGA, LPC, and CCNE1 copy number profile to derive an HRD score.	LGA + LPC + CCNE1 CN	Proprietary threshold	Not available	>20% tumor cellularity, surface tumoral content above 10 mm^2^	Not available	CE-IVDR
NOGGO GIS	Charite Berlin and University of Hamburg	BRCA1, BRCA2 and 55 HRR genes	20,000 SNPs are analyzed to calculate NOGGO genomic instability score.	LOH + CNA + Telomeric CNA	83–85 (vs. Myriad)	Not available	40 ng of DNA	Yes	LDT
Leuven HRD	University of Leuven	BRCA1, BRCA2, RAD51C, RAD51D, PALB2, BLM, BARD1, BRIP1 and TP53	90,000 SNPs are analyzed to determine the genomic instability score.	GIS (LOH + TAI + LST)	GIS > 56	Not available	50–100 ng of DNA	Yes	LDT
Hospital Geneva HRD test	Geneva University	Only BRCA1, BRCA2 for ovarian cancer	Copy number alterations are analyzed the normalized large-scale transition score.	nLST	≥15	Not available	100 ng od DNA, 20% minimum tumor content	Yes	LDT

List of acronyms used in the table: SNPs: small nucleotide polymorphisms, CNVs: copy number variations, HRD: homologous recombination deficiency, HRR: homologous recombination repair, sWGS: shallow whole genome sequencing, LOH: loss of heterozigosity, TAI: telomeric allelic imbalances, LST: large-scale transitions, nLST: normalized large-scale transitions, LGA: Large Genomic Alterations, LPC: Loss of Parental Copy, GIS: genomic instability score, GIM: genomic instability metric, GSS: genomic scar score, GII: genomic instability index, BRCA1, BRCA2, CCNE1, RAD51C, RAD51D, PALB2, BLM, BARD1, BRIP1, TP53: genes. LDT: Laboratory-Developed Test, CE-IVD: European Conformity In Vitro, FDA: Food and Drug Administration.

## Data Availability

The data presented in this study are available upon request from the authors.
